# Synergistic Interaction of Dietary Pattern and Concordance Lifestyle with Abnormal Liver Function among Young Adults in Taiwan: A Population-Based Longitudinal Study

**DOI:** 10.3390/nu13103591

**Published:** 2021-10-14

**Authors:** Rathi Paramastri, Chien-Yeh Hsu, Yung-Kun Chuang, Hsiu-An Lee, Bayu Satria Wiratama, Jane C.-J. Chao

**Affiliations:** 1 School of Nutrition and Health Sciences, College of Nutrition, Taipei Medical University, 250 Wu-Hsing Street, Taipei 11031, Taiwan; rara.paramastri@gmail.com; 2 Department of Information Management, National Taipei University of Nursing and Health Sciences, 365 Ming-Te Road, Peitou District, Taipei 11219, Taiwan; cyhsu@ntunhs.edu.tw; 3 Master Program in Global Health and Development, College of Public Health, Taipei Medical University, 250 Wu-Hsing Street, Taipei 11031, Taiwan; 4 Master Program in Food Safety, College of Nutrition, Taipei Medical University, 250 Wu-Hsing Street, Taipei 11031, Taiwan; ykchuang@tmu.edu.tw; 5 School of Food Safety, College of Nutrition, Taipei Medical University, 250 Wu-Hsing Street, Taipei 11031, Taiwan; 6 Nutrition Research Center, Taipei Medical University Hospital, 252 Wu-Hsing Street, Taipei 11031, Taiwan; 7 National Health Research Institutes, 35 Keyan Road, Zhunan Town, Miaoli 350, Taiwan; billy72325@gmail.com; 8 Department of Epidemiology and Biostatistics, Faculty of Medicine Public Health and Nursing, Universitas Gadjah Mada, Yogyakarta 55281, Indonesia; bayu.satria@ugm.ac.id

**Keywords:** dietary pattern, smoking, alcohol drinking, sleep, physical activity, body mass index, liver function, adults, reduced rank regression

## Abstract

While diet and lifestyle are independently implicated in the etiology of liver disease, the interaction of diet and lifestyle may be more helpful for determining the risk of liver abnormality. Thus, our study aimed to evaluate the interaction between the dietary pattern associated with liver biomarkers and lifestyle factors among Taiwanese adults with abnormal liver enzymes. A liver-associated dietary pattern, generated using reduced rank regression, was characterized by high intake of soy sauce or other dips, sugar sweetened beverages, and preserved and processed foods, but low intake of seafood, fruits, eggs, and dark-colored vegetables. In the fully adjusted model, liver-associated dietary patterns or unhealthy concordance lifestyle factors were associated with an increased risk of having liver function abnormality (OR = 1.08, 95% CI: 1.04, 1.12 and OR = 1.42, 95% CI: 1.31, 1.53, respectively). Moreover, the interaction between liver-associated dietary pattern and unhealthy concordance lifestyle factors showed more significant correlation, with an elevated risk of abnormal liver function (OR = 2.14, 95% CI: 2.02, 2.26). Therefore, our study suggests that participants who have a strong liver-associated dietary pattern along with unhealthy concordance lifestyles are likely to have increased odds of abnormal liver function.

## 1. Introduction

Liver disease represents the hepatic manifestation of complex metabolic symptoms [1] and often coexists with obesity, dyslipidemia, and insulin resistance [2]. Evaluating the hepatic dysfunction includes the diagnosis of hepatocyte injury and structural liver disease based on signs, symptoms, and biochemical measures of serum biomarkers [3,4]. Serum hepatic enzymes such as alanine transaminase (ALT), aspartate transaminase (AST), and gamma-glutamyltransferase (γ-GT) have been used as parameters for diagnosing defects in liver function [5]. Several conditions of damaged liver can be identified by the elevation of serum ALT, AST, and γ-GT [6,7].

The emerging liver disorder commonly found in adults is nonalcoholic fatty liver disease (NAFLD). Globally, the estimated prevalence of NAFLD is 24% in the general population, with the highest number acquired from South America and the Middle East, followed by Asia and Europe [8]. Among individuals aged ≥20 years, the prevalence of NAFLD ranged from 20–30% in the Hong Kong, South Korea, and Taiwan populations in 2015 [9]. A previous study using Markov modelling to project disease burden reported that the prevalence of NAFLD was estimated to rise from 6 to 20% between 2019 and 2030; likewise, the mortality rate of NAFLD was also projected to increase from 65 to 100% [9]. The previous study suggested that increased incidence of NAFLD in the Asia-Pacific region was ascribed to the latest adaptation of Westernized dietary and lifestyle habits [9]. Correspondingly, unhealthy dietary habits and sedentary lifestyle have been adopted rapidly in a habitual lifestyle among Taiwan’s population, and the risk of chronic disease is expected to increase [10].

Dietary habits have been considered as a potential environmental factor in relation to liver disease. A study among adults in Taiwan showed that individuals in the highest quartile of a Western dietary pattern, characterized by animal protein, eggs, refined foods and beverages, oil-cooked foods, and high-sugar foods, were likely to have elevated ALT levels (OR = 1.43, 95% CI: 1.04, 1.97), while individuals consuming the Mediterranean diet were correlated with a reduced risk of having abnormal levels of γ-GT (OR = 0.72, 95% CI: 0.53, 0.97) [11]. In accordance, a previous investigation among Iranian adults found greater odds of having NAFLD in subjects with the highest adherence to a Western dietary pattern (OR = 2.61, 95% CI: 1.41, 4.28); meanwhile, subjects in the highest quartile of a healthy dietary pattern were 41% less likely to have NAFLD after controlling for confounders [12]. Moreover, a previous report also mentioned that the role of sedentary lifestyle or physical inactivity was independent from the progression of liver disease [13]. A review investigation of 20 observational studies revealed that the pooled odds ratio for the association between actively smoking and NAFLD was 1.11 (95% CI: 1.03, 1.19) [14]. Therefore, both dietary habits and lifestyles were suggested to be potentially associated with liver dysfunction.

Despite investigations evaluating the roles of dietary habits and lifestyle variables in chronic disease progression, very few studies have aimed to explore the association between them. Because dietary habits and lifestyles cannot be separated from one another in terms of daily activities, such an investigation is crucial for understanding the interaction of dietary habits and lifestyles on liver dysfunction. Many studies have focused on related aspects, such as nutrition and physical activity, while neglecting other modes of social behavior. Thus, our objective was to investigate the interaction between dietary patterns associated with liver biomarkers and lifestyle factors associated with abnormal serum liver enzymes among Taiwanese adults in order to take a step toward preventing liver disease. Moreover, to the best of our knowledge, our study is the first one to investigate the synergistic effect of dietary patterns and lifestyle habits on liver dysfunction in Taiwan.

## 2. Materials and Methods

### 2.1. Study Cohort and Design

Our study was a longitudinal study using data from Mei Jau (MJ) database, which contained data from a prospective study of Taiwanese populations who participated in multiple comprehensive health screening examinations. Prior to enrollment, all participants had read, understood, and signed the informed consent forms provided by MJ Health Management Institution; the study collected no personal identification and information was used for research purposes only. Information on MJ Health Screening Centers has been clearly defined in previous studies [15,16]. Participants completed a self-reported questionnaire related to sociodemographic data, lifestyle habits, history of chronic diseases, and dietary consumption at baseline. Participants also underwent physical examination and provided biological samples for laboratory testing in accordance with ISO 9001 quality management standards.

Initially, we retrieved 190,200 participants from the MJ database from 2001 to 2015. From this, we randomly recruited participants who met the following criteria: (1) aged between 20 and 45 years, (2) free of chronic diseases, (3) having normal levels of serum liver enzymes at baseline and no history of receiving hepatic treatment, (4) having at least one follow-up visit, and (5) having complete data at baseline. Finally, we included 62,645 participants for analysis. The study design was approved by Taipei Medical University—Joint Institutional Review Board (TMU-JIRB N202001055).

### 2.2. Socio-Demographic Characteristics and Lifestyle Factors

Information related to socio-demographic characteristics and lifestyle factors was collected using a self-reported questionnaire or obtained via face-to-face interviews by trained medical professionals. The collected socio-demographic information included age, gender, marital status, education, and family income. Lifestyle factors, including smoking status, alcohol drinking, sleep habits, and physical activity, were divided into certain categories, which were clearly described in a previous study [17]. We categorized each lifestyle factor into quartiles, and the higher quartile represented the higher degree of unhealthy lifestyle. Moreover, we combined smoking status, drinking status, sleep duration, and physical activity as one group of concordance lifestyle, which was also divided into quartiles to indicate the degree of unhealthy lifestyle.

### 2.3. Anthropometric, Biochemical, and Clinical Data

The assessment of anthropometric measurements, including body weight (kg), height (cm), and body fat (%), was performed in MJ Health Screening Centers. Body weight and height were assessed using electronic scales and an auto-anthropometer (KN-5000A, Nakamura, Tokyo, Japan). Body mass index (BMI) was calculated as weight (kg) divided by the square of height (m^2^) and further classified into underweight (BMI < 18.5 kg/m^2^), normal weight (18.5 kg/m^2^ ≤ BMI < 24 kg/m^2^), overweight (24 kg/m^2^ ≤ BMI < 27 kg/m^2^), and obese (BMI ≥ 27 kg/m^2^) categories according to Taiwan’s criteria [18]. The body fat percentage of participants was measured using a bioelectrical impedance analysis instrument (InBody Co., Ltd., Seoul, Korea).

All blood samples and blood pressure were taken at MJ Health Screening Centers after 12–14 h overnight fasting. The MJ laboratory was responsible for all measurements of blood sample tests. Because we did not obtain further diagnostic information on liver disease, such as the infection of hepatotoxic viruses, liver biopsy, or liver echogenicity, from the MJ database, and serum hepatic enzymes such as ALT, AST, and γ-GT are frequently determined in most health screening facilities and hospitals in Taiwan, we used the enzymatic methodology to evaluate liver function. Our study applied the identification of abnormal liver function based on the abnormal ranges of liver enzymes offered by the MJ laboratory as follows: ALT > 33 U/L, AST > 27 U/L, or γ-GT ≥ 39 U/L for women and γ-GT ≥ 50 U/L for men [11,19]. Our study also retrieved data corresponding to iron biomarkers, such as red blood cells (RBC, ×10^6^/μL), hemoglobin (mmol/L), hematocrit (%), mean corpuscular volume (MCV, fL), mean corpuscular hemoglobin (MCH, pg), mean corpuscular hemoglobin concentration (MCHC, g/dL), iron (μg/dL), and ferritin (ng/mL).

### 2.4. Dietary Assessment

Dietary data were obtained using a standardized and validated semi-quantitative food frequency questionnaire (SQ-FFQ) designed by MJ Health Management, which contained 22 food items generated from 85 closed-ended questions on dietary habits [15,16]. The frequency of consuming a fixed serving daily or weekly in the previous month prior to data collection was answered by self-reporting. Each food item question had 5 response options for the frequency of consumption, as mentioned previously [15,20]. From the dietary data of the SQ-FFQ, we derived the dietary pattern using a reduced rank regression approach, which combined a priori and exploratory statistical methods [21]. For generating the dietary pattern associated with liver function, 7 response variables, including ALT, AST, γ-GT, alkaline phosphatase (ALP), lactate dehydrogenase (LDH), albumin, and total bilirubin, were applied, and 22 food items were used as the predictors. We only retracted food items with absolute factor loadings ≥ 0.20 to obtain the most representative dietary habits of study participants [22,23]. The scores of the derived dietary patterns were classified into quartiles, and the higher quartile of the derived dietary pattern, the higher the risk for abnormal liver enzymes.

### 2.5. Statistical Analysis

The current study described the distribution of demographic characteristics, lifestyles, clinical data, and biochemical measurements at baseline and follow-up. The characteristics of participants were classified as “normal” and “abnormal” liver function. We examined the normality of data using the Kolmogorov-Smirnov test [24]. The chi-square test for categorical data and Wilcoxon rank-sum test for continuous data were performed to compare the differences between normal and abnormal liver function groups. Furthermore, we performed multinomial logistic regression to predict the risk of abnormal liver function, and factors such as age, gender, family income, education, BMI, body fat, blood pressure, and iron biomarkers, including hemoglobin, hematocrit, iron, and ferritin, were controlled in different models. We estimated multivariate analysis with the adjustment of age and gender in model 1. Moreover, we added marital status, education, family income, BMI, body fat, systolic and diastolic pressure, smoking status, drinking status, sleep duration, physical activity, and iron biomarkers in model 2, except for the factors in model 1, for analysis of the association between liver-associated dietary patterns and the risk of abnormal liver function. For investigation of the association between concordance lifestyle and the risk of abnormal liver function, similar factors without lifestyle factors were adjusted in model 2. Additionally, to assess the potential interaction between liver-associated dietary patterns and concordance lifestyle stratified across the quartiles, we conducted a joint/combined analysis of dietary pattern and concordance lifestyle using the Strobe approach [25], where the reference group was participants in the first quartile of dietary pattern or concordance lifestyle scores. Therefore, we generated 16 combinations between dietary pattern and concordance lifestyle to represent the interaction. Our study performed sensitivity analysis using multivariate linear regression to derive the linear relationship between serum liver biomarkers and quartiles of dietary pattern [26]. All analyses were conducted using STATA version 13 (StataCorp LP, College Station, TX, USA). Significant differences were set using α = 5%.

## 3. Results

### 3.1. Characteristics of Study Participants

In the longitudinal observation from 2001 to 2015, 11,506 participants developed abnormal liver function (18.4%). Our data revealed that after the participants exhibited altered liver enzymes, this condition remained until the last observation. The demographic and lifestyle characteristics of participants at baseline and follow-up, stratified by liver function, are reported in Table 1. At baseline, the majority of participants were female (78.8%), married (62.5%), non-smokers (82.9%), and non-drinkers (92.4%), had education equivalent to high school or above (83.6%), had a family income < 800,000 NTD (60.4%), had a 6–8 h sleep duration (71.1%), and performed physical activity ≤ 2 h/week (77.3%). Furthermore, these characteristics remained after follow-up observation in both groups. In addition, we found significant differences in the mean of the observational period between the two groups. The participants who experienced abnormal liver function had a shorter observational period than those with normal liver function (4.1 ± 2.7 vs. 4.8 ± 3.2 years).

The clinical and biochemical data of study participants classified by liver function are given in Table 2. Participants with abnormal liver function had higher proportions of overweight (22.4% vs. 13.8%) and obesity (10.5% vs. 5.5%) compared to those with normal liver function. In addition, body fat, blood pressure, all liver function biomarkers, and all iron biomarkers were significantly higher in participants with abnormal liver function.

### 3.2. Liver-Associated Dietary Pattern and Liver Function Biomarkers

A dietary pattern closely related to liver abnormality was identified in this study using a reduced rank regression (RRR) model. We named the dietary pattern “liver-associated dietary pattern” (Table 3). Dipping sauces, sugar-sweetened beverages, and preserved and processed foods were positively associated with liver-associated dietary patterns (factor loading > 0.20). On the contrary, seafood, fruits, eggs, and dark-colored vegetables showed negative correlations with the observed dietary pattern (factor loading < −0.20, |factor loading| > 0.20). The cumulative percentage of variation explained by the observed dietary pattern was 6.87%, and six response variables, including ALT, AST, ALP, LDH, albumin, and total bilirubin, explained 3.7% of the total variation (data not shown). Our study found significant correlations between the quartiles of dietary patterns and six of seven serum liver function biomarkers, except for γ-GT, after controlling for age, gender, marital status, education, family income, BMI, body fat, blood pressure, and lifestyle variables (Supplementary Table S1). Positive correlations were observed between liver-associated dietary patterns and the levels of ALT (β = 0.55, 95% CI: 0.42, 0.68), AST (β = 0.47, 95% CI: 0.26, 0.68), ALP (β = 12.76, 95% CI: 11.90, 13.62), LDH (β = 13.59, 95% CI: 12.59, 14.58), and total bilirubin (β = 0.01, 95% CI: 11.90, 0.01, 0.02). On the other hand, the highest quartile of liver-associated dietary pattern was inversely correlated with albumin levels (β = −0.02, 95% CI: −0.02, −0.01).

### 3.3. Interactive Association of Dietary Pattern, Lifestyles, and Liver Function Biomarkers

Our study observed the higher odds of experiencing abnormal liver function biomarkers for participants who smoked, drank alcohol, had abnormal sleep duration (<6 h or >8 h), did less physical activity (<2 h/week), and had abnormal BMI status in the fully adjusted model (Supplementary Table S2). However, second-hand smokers and past drinkers had no association with abnormal liver function. We found that participants who smoked or drank on a daily basis were correlated with higher odds of developing liver abnormality by 54% (95% CI: 1.47, 1.61) and 57% (95% CI: 1.38, 1.78), respectively, compared to those who were non-smokers or non-drinkers. Moreover, participants who had less physical activity were 25% more likely to have higher odds of abnormal liver function (95% CI: 1.17, 1.33) compared to those who had more active physical activity (>2 h/week). Participants with underweight, overweight, or obesity were associated with much higher odds of abnormal liver function, by 71% (95% CI: 1.62, 1.80), 215% (95% CI: 2.96, 3.39), and 352% (95% CI: 4.23, 4.85), respectively, compared to those with normal weight.

The association of dietary pattern across the quartiles and concordance lifestyle with the risk of abnormal liver function is presented in Table 4. Both higher quartiles of liver-associated dietary patterns and concordance lifestyle were positively correlated with higher odds of abnormal liver function. In model 2, participants in the highest quartile of liver-associated dietary pattern or concordance lifestyle were associated with higher odds of abnormal liver function of 8% (95% CI: 1.04, 1.12) and 42% (95% CI: 1.31, 1.53), respectively, compared to those in the corresponding reference group. Furthermore, we observed the association of different proportions of dietary or lifestyle exposure with abnormal liver enzymes in both genders (Supplementary Table S3). Male participants in the highest quartile of liver-associated dietary patterns had increased odds of abnormal liver function by 13% compared to those in the lowest quartile. Female participants in higher quartiles of liver-associated dietary pattern had higher odds of abnormal liver function by 9–18%. Male participants who were past smokers but not current smokers had greater odds of abnormal liver function, while female participants who were past or current smokers had higher odds of abnormal liver function. Male participants who were past or current drinkers had increased odds of abnormal liver function, while female participants who were frequent drinkers (≥3 times/week) had greater odds of abnormal liver function. Male participants who had long sleep duration had higher odds of abnormal liver function, while female participants who had short or long sleep duration had greater odds of abnormal liver function. Male and female participants who had less physical activity had increased odds of abnormal liver function by 31% (95% CI: 1.10, 1.55) and 24% (95% CI: 1.03, 1.48), respectively.

Moreover, the interaction between liver-associated dietary patterns and concordance lifestyle for the risk of abnormal liver function is shown in Table 5. Individuals who were in both the highest quartile of liver-associated dietary patterns and concordance lifestyle were more likely to have increased odds of developing liver function abnormality, by 114% (95% CI: 2.02, 2.26), compared to those who were in both the lowest quartile of liver-associated dietary pattern and concordance lifestyle.

## 4. Discussion

Our study generated interactive models and determined the effects of joint exposure of liver-associated dietary patterns and concordance lifestyle on developing abnormal liver function. This study revealed that individuals with poor lifestyle behaviors such as smoking, alcohol consumption, abnormal sleep duration, and less physical activity were associated with higher odds of developing abnormal liver function. These associations were independent of age, gender, marital status, education, family income, BMI, body fat, blood pressure, and iron biomarkers. Correspondingly, participants who were in higher quartiles of the reduced rank regression–derived dietary pattern, namely the liver-associated dietary pattern, were significantly associated with disease progression. Interestingly, higher quartiles of dietary pattern and lifestyle interaction were prone to liver dysfunction. To our knowledge, this study provided the first evidence that the interplay of poor dietary pattern and lifestyle behaviors might worsen liver function outcomes in a longitudinal setting.

Previous studies showed a positive correlation of poor lifestyle behaviors with liver dysfunction [14,27,28,29]. Accordingly, our investigation revealed that individuals who were exposed to poor lifestyle behaviors were associated with abnormal liver function biomarkers, which indicated a higher risk of developing liver dysfunction. The liver-associated dietary pattern showed a correlation with higher odds of developing abnormal liver function. The characteristics of the derived dietary pattern, with high consumption of dipping sauces, sugar-sweetened beverages, and processed foods but low consumption of presumably healthy food, such as seafood, fruits, eggs, and dark vegetables, were similar to an unhealthy dietary pattern and could be associated with an increased risk of developing abnormal liver function [30]. A previous investigation among the Taiwanese population indicated that Western dietary patterns, known as unhealthy diets, appeared to be a risk factor in developing abnormal serum ALT levels in female participants but not in male participants [11]. In gender-specific analyses, we observed that the role of dietary patterns on disease progression occurred across quartiles in female participants, while only the highest quartile of dietary pattern showed a significant association with abnormal liver function in male participants. Previous evidence suggests that an unhealthy dietary pattern, mostly consisting of high-fat and high-energy foods, is closely related to liver disease; however, only a few studies have examined dietary patterns with greater adherence to refined foods and sugary drinks or high-energy but low-nutrient-density foods in NAFLD progression [31,32]. Interestingly, we found a significant correlation between the development of liver abnormality and the dietary pattern characterized by high sodium foods and sweetened beverages. Dipping sauces and processed foods consumed in the liver-associated dietary pattern contain high sodium, which might be related to the development of liver disease [30,33]. In a cross-sectional study among 100,177 participants in Korea, the dietary pattern with high sodium intake, including sauces and processed meals, was significantly associated with a greater prevalence of NAFLD (PR = 1.25, 95% CI: 1.18, 1.32 in men and PR = 1.32, 95% CI: 1.18, 1.47 in women) [30]. In addition, a cross-sectional study among NAFLD patients demonstrated a positive association between the dietary pattern characterized by refined grains, fried foods, meat, and processed meat and the presence of NAFLD [33]. Despite the fact that the mechanism for the effect of sodium on liver disease is not completely understood, an experimental study conducted in rats suggested that sodium could have a direct effect on obesity through inducing the hypertrophy of adipocytes by improving insulin sensitivity and glucose metabolism stimulated by insulin in the adipocytes [34]. In agreement with our study, the results revealed that overweight and obesity were positively correlated with a greater increased risk of abnormal liver function.

Moreover, individuals who adhered to fast foods or sugar sweetened beverages (3–5 times/week) were more likely to increase serum ALT (OR = 1.27, 95% CI: 1.05, 1.54 and OR = 1.24, 95% CI: 1.07, 1.42, respectively) compared to those who did not consume these foods among Korean overweight or obese teenage students [35]. Additionally, a randomized intervention study among 47 overweight or obese subjects aged 20–50 years suggested that subjects who consumed 1 L of sugar-containing drinks daily for 6 months significantly increased liver and visceral fat [36]. A systematic review and meta-analysis of seven studies (six cross-sectional studies and one cohort study) involving 4639 participants reported that sugar-sweetened beverage consumers were more likely to be correlated with an increased risk of developing NAFLD of 53% (RR = 1.53, 95% CI: 1.34, 1.75, *I*^2^ = 0%) compared to non-consumers [37]. Some reports suggest that sweeteners such as fructose and sucrose in soft drinks and snacks could stimulate lipogenesis and inhibit mitochondrial β-oxidation of fatty acids, which leads to increases in liver fat and serum liver enzyme levels [32,38]. Nutrient components of the healthy dietary pattern, such as dietary fiber, vitamins, and minerals, were not abundant in our derived liver-associated dietary pattern, which could plausibly suggest that an increased risk of developing liver abnormality was found among the participants following the liver-associated dietary pattern.

Regarding the association of smoking and abnormal liver function, a review study among 20,149 individuals aged above 18 years supported a positive relationship between smoking and NAFLD (pooled OR = 1.110, 95% CI: 1.028, 1.199) [14]. In addition, a population-based study in China demonstrated higher odds for NAFLD among individuals who were former smokers (OR = 1.45, 95% CI: 1.05, 2.00) or current heavy smokers (OR = 2.29, 95% CI: 1.30, 4.03) compared to non-smokers [39]. Moreover, previous studies have also observed the independent and combined effects of active cigarette smoking and alcohol consumption on an elevation of serum liver enzyme levels [40,41]. An epidemiological cohort study among middle-aged and elderly subjects in Germany showed that individuals who were combined heavy smokers (≥21 cigarettes/d) and moderate to heavy drinkers (≥100 g alcohol/week) were associated with an increased odds of elevated γ-GT to 2.9 (95% CI: 1.1, 7.6) and to 3.8 (95% CI: 2.2, 6.6) in women and men, respectively, compared to non-smokers and non-drinkers [40]. Another study also supported this finding: a population-based prospective cohort study conducted among 5946 adults in rural and urban areas of South Korea observed that combined heavy cigarette smoking (>20 pack years) and heavy alcohol consumption (≥24 g/d) showed a supra-additive effect with synergic index (SI) > 1 on an elevation of γ-GT (SI = 2.03, 95% CI: 1.75, 2.35) and AST levels (SI = 4.55, 95% CI: 3.12, 6.61) [41]. This association could be addressed by the assumption that both smoking and alcohol consumption might stimulate oxidative stress and induce the depletion of anti-inflammatory substances such as glutathione in various tissues, including the liver [40,42]. Likewise, a rat study revealed that oxidative stress in the liver induced by chronic combined administration of alcohol and nicotine could lead to additive oxidative damage, such as lipid peroxidation in the liver [42].

The association between sleep duration and an increased risk of liver abnormalities has been widely investigated in epidemiologic studies, although the results remain inconclusive. Interestingly, our study reported that both short and long sleep duration played a role in developing liver abnormalities. A meta-analysis study including 59,094 participants found an increased risk of having NAFLD among participants who had short sleep duration compared to those who had longer sleep duration (pooled RR = 1.19, 95% CI: 1.04, 1.36, *I*^2^ = 0%) [43]. A population-based longitudinal study among 12,306 Japanese revealed that short sleep duration ≤ 5 h in both women (HR = 1.46, 95% CI: 1.05, 2.04) and men (HR = 1.39, 95% CI: 1.13, 1.72) was significantly correlated with an increased risk of developing NAFLD compared to longer sleep duration (>7 h) after follow-up for 6.8 (for men) or 7 (for women) years [44]. Previous reports also suggest that dietary intake might have a mediating effect on the association between sleep duration and liver abnormalities [45,46]. Thus, a link between short sleep duration (≤6 h) and greater calorie intake has been observed as compared to normal sleep duration (7 h or 7–8 h) [45,46], and individuals who had short sleep duration (5–6 h) had a tendency toward increased carbohydrate, sugar, and fat consumption compared to those who had normal sleep duration (7–8 h) [46]. As a result, short sleep duration could be accompanied by changes in appetite-related hormones, eating behavior, and nutrient metabolism, which might further raise the risk of certain chronic diseases such as liver disease [47].

Evidence supporting our findings has shown that less physical activity was associated with aberrant liver function. A cross-sectional study conducted among 139,056 middle-aged Koreans reported an inverse association between physical activity and NAFLD (PR = 0.80–0.94, 95% CI: 0.78–0.92, 0.82–0.95) [48]. Furthermore, Ryu et al. [48] also found that participants who were physically inactive tended to have unhealthy dietary patterns. Subjects with prolonged sitting times were more likely to be associated with an increased calorie intake, by 36% (95% CI: 1.31, 1.43) [48]. This finding was consistent with the results from a small-scale cross-sectional study of 74 British adults, which found that NAFLD individuals had approximately an additional half-hour per day of sedentary behavior (1318 ± 68 vs. 1289 ± 60 min/day, *p* < 0.05) and walked fewer steps (8483 ± 2926 vs. 10,377 ± 3529 steps/day, *p* < 0.01) compared to the healthy controls [29]. Adopting sedentary behaviors over a long period of time could limit overall energy expenditure by muscle contraction and impair lipoprotein lipase activity, which leads to the inhibition of triglyceride uptake by skeletal muscles and impedes fat metabolism in peripheral tissues; thus, the role of sedentary behavior might indirectly affect fat metabolism in the liver and further be related to the etiology of NAFLD [29,49].

With respect to the significant association between concordance lifestyle behavior and abnormal liver function, we found that joint lifestyle behaviors played a more significant role in the presence of abnormal liver function than dietary pattern did. This finding could be explained by the fact that combined lifestyle habits can have more significant effects on improving or worsening the disease of interest [50]. A previous study demonstrated that an unhealthy dietary pattern was correlated with a higher risk of NAFLD, especially among obese participants [51]. Poor lifestyle habits, such as a lack of exercise, exacerbated abnormal liver function, independently of body weight. As matter of fact, our participants were more likely to have normal weight status, and this could explain the limited association of the dietary pattern with the development of liver abnormalities.

A major strength of our study was the large sample size, providing a reliable statistical power to evaluate the longitudinal data. Because liver disease is caused by multifactorial exposure [52], we expanded our analysis to observe the combined exposures of dietary pattern and lifestyle factors in the development of disease outcomes. The novelty of exploring the synergistic correlation between four-component evolutionary concordance lifestyle and dietary pattern-specific liver abnormality was also our notable strength. Moreover, our study was the first study to conduct such an interaction analysis in Taiwan. As a result, our findings might make a significant contribution to elucidate the effect of various lifestyle variables on the prevention and management of liver abnormalities. However, there are some limitations that should be mentioned. First, the histological definition of liver disease, such as through biopsy, was not included because invasive procedures were not acceptable in a population-based epidemiological investigation [53]. Therefore, we described the study participants with normal or abnormal liver function according to serum liver enzyme levels such as ALT, AST, and γ-GT [5]. Second, because lifestyle habits, including smoking, alcohol drinking, sleeping habits, and physical activity, were self-reported, misclassification was inevitable. Third, the well-known limitations of SQ-FFQ, including recall errors, a limited number of food items, and difficulties in estimating intake frequencies, were also present in this study [54].

## 5. Conclusions

Our findings suggest that individuals who followed the liver-associated dietary pattern alone or combined with a concordance lifestyle are positively associated with abnormal liver function among adults in Taiwan. Despite these findings of epidemiological investigation, the prospective cohort study of the interaction between several lifestyle behaviors in the development of liver disease is required.

## Figures and Tables

**Table 1:** Demographic and lifestyle characteristics of study participants classified by liver function (*n* = 62,645) ^a^.

Variables	Baseline ^b^	Follow-Up ^b^		
	Total (*n* = 62,645)	Normal Liver Function (*n* = 51,139)	Abnormal Liver Function ^c^ (*n* = 11,506)	*p*-Value
Age (years)	37.6 ± 12.6	38.0 ± 13.5	42.5 ± 13.4	<0.0001
Sex				<0.0001
Male	13,189 (21.1%)	9223 (18.0%)	3966 (34.5%)	
Female	49,456 (78.8%)	41,916 (82.0%)	7540 (65.5%)	
Observational period (years)		4.8 ± 3.2	4.1 ± 2.7	<0.0001
Marital status				<0.0001
No	23,519 (37.5%)	19,705 (38.5%)	3814 (33.1%)	
Married	39,126 (62.5%)	31,434 (61.5%)	7692 (66.9%)	
Education				<0.0001
<High school	10,277 (16.4%)	8144 (15.9%)	2133 (18.5%)	
≥High school	52,368 (83.6%)	42,995 (84.1%)	9373 (81.5%)	
Family income				0.247
<800,000 NTD	41,417 (60.4%)	33,863 (66.2%)	7554 (65.7%)	
≥800,000 NTD	21,228 (39.6%)	17,276 (33.8%)	3952 (34.3%)	
Smoking status				<0.0001
Non-smoker	51,948 (82.9%)	42,857 (83.8%)	9091 (79.0%)	
Second-hand smoker	3635 (5.8%)	2982 (5.8%)	653 (5.7%)	
Past smoker	1470 (2.4%)	1147 (2.2%)	323 (2.8%)	
Smoke occasionally	1339 (2.1%)	915 (1.8%)	424 (3.7%)	
Smoke daily	4253 (6.8%)	3238 (6.4%)	1015 (8.8%)	
Drinking status				<0.0001
Non-drinker	57,871 (92.4%)	47,591 (93.1%)	10,280 (89.3%)	
Past drinker	570 (0.9%)	444 (0.9%)	126 (1.1%)	
1–2 times/week	3096 (4.9%)	2295 (4.5%)	801 (6.9%)	
3–4 times/week	768 (1.2%)	551 (1.1%)	217 (1.9%)	
Drank daily	340 (0.6%)	258 (0.4%)	82 (0.8%)	
Sleep duration				<0.0001
Short (<6 h)	12,767 (20.4%)	10,255 (20.1%)	2512 (21.8%)	
Normal (6–8 h)	44,562 (71.1%)	36,455 (71.3%)	8107 (70.5%)	
Long (>8 h)	5316 (8.5%)	4429 (8.6%)	887 (7.7%)	
Physical activity				<0.0001
Less active (≤2 h/week)	48,404 (77.3%)	40,006 (78.2%)	8398 (72.9%)	
Active (>2 h/week)	14,241 (22.7%)	11,133 (21.8%)	3108 (27.1%)	

^a^ Continuous data are presented as mean ± SD, and categorical data are presented as number (percentage). The *p*-value was analyzed using the Mann-Whitney U test for continuous variables and the chi-square test for categorical variables. ^b^ Percentage was calculated within the same group. ^c^ Abnormal liver function was defined as ALT > 33 U/L, AST > 27 U/L, or γ-GT ≥ 39 U/L for women and γ-GT ≥ 50 U/L for men.

**Table 2:** Clinical and biochemical data of study participants classified by liver function (*n* = 62,645) ^a^.

Variables	Baseline ^b^	Follow-Up ^b^		
	Total (*n* = 62,645)	Normal Liver Function (*n* = 51,139)	Abnormal Liver Function ^c^ (*n* = 11,506)	*p*-Value
Body mass index (kg/m^2^) ^d^				<0.0001
Underweight	8463 (13.5%)	7658 (14.9%)	805 (6.9%)	
Normal weight	40,536 (64.7%)	33,619 (65.7%)	6917 (60.1%)	
Overweight	9642 (15.4%)	7070 (13.8%)	2572 (22.3%)	
Obese	4004 (6.4%)	2792 (5.6%)	1212 (10.7%)	
Body fat (%)	26.6 ± 6.7	26.6 ± 6.9	29.6 ± 7.8	<0.0001
Systolic pressure (mmHg)	113.4 ± 7.4	113.9 ± 6.9	122.8 ± 7.6	<0.0001
Diastolic pressure (mmHg)	69.4 ± 1.8	68.0 ± 1.3	74.8 ± 1.9	<0.0001
Liver function biomarkers				
ALT (U/L)	24.3 ± 2.6	17.6 ± 7.0	50.6 ± 4.6	<0.0001
AST (U/L)	22.3 ± 4.6	19.3 ± 4.2	34.0 ± 8.5	<0.0001
γ-GT (U/L)	21.5 ± 2.5	14.6 ± 5.6	48.7 ± 4.5	<0.0001
ALP (U/L)	94.27 ± 6.4	93.07 ± 6.3	99.0 ± 4.7	<0.0001
LDH (U/L)	209.4 ± 7.9	207.5 ± 7.7	216.9 ± 8.7	<0.0001
albumin (g/dL)	4.5 ± 0.2	4.4 ± 0.2	4.5 ± 0.3	<0.0001
Total bilirubin (mg/dL)	0.8 ± 0.3	0.8 ± 0.3	0.9 ± 0.3	<0.0001
Iron biomarkers				
RBC (×10^6^/μL)	4.7 ± 0.5	4.7 ± 0.4	4.9 ± 0.4	<0.0001
Hemoglobin (mmol/L)	8.6 ± 0.9	8.5 ± 0.8	9.0 ± 0.9	<0.0001
Hematocrit (%)	41.1 ± 4.1	40.6 ± 4.0	43.3 ± 4.2	<0.0001
MCV (fL)	87.9 ± 6.7	87.7 ± 6.8	88.6 ± 6.2	<0.0001
MCH (pg)	29.5 ± 2.7	29.4 ± 2.7	29.8 ± 2.5	<0.0001
MCHC (g/dL)	33.6 ± 0.8	33.5 ± 0.8	33.6 ± 0.8	<0.0001
Iron (μg/dL)	92.9 ± 3.7	90.8 ± 3.7	101.4 ± 3.7	<0.0001
Ferritin (ng/mL)	125.6 ± 1.1	107.7 ± 1.4	214.6 ± 1.6	<0.0001

ALT: alanine transaminase, AST: aspartate transaminase, γ-GT: gamma-glutamyltransferase, ALP: alkaline phosphatase, LDH: lactate dehydrogenase, RBC: red blood cells, MCV: mean corpuscular volume, MCH: mean corpuscular hemoglobin, MCHC: mean corpuscular hemoglobin concentration. ^a^ Continuous data are presented as mean ± SD, and categorical data are presented as number (percentage). The *p*-value was analyzed between the participants with normal and abnormal liver function during follow-up using the Mann-Whitney U test for continuous variables and the chi-square test for categorical variables. ^b^ Percentage was calculated within the same group. ^c^ Abnormal liver function was defined as ALT > 33 U/L, AST > 27 U/L, or γ-GT ≥ 39 U/L for women and γ-GT ≥ 50 U/L for men. ^d^ The values of BMI were categorized into underweight (<18.5 kg/m^2^), normal weight (18.5–23.9 kg/m^2^), overweight (24.0–26.9 kg/m^2^), and obese (≥27 kg/m^2^).

**Table 3:** Percentage of variation explained by liver-associated dietary patterns and factor loadings of 22 food items by reduced rank regression analysis.

Food Items	Explained Variation (%)	Factor Loading
Soy sauce or other dips	45.9	0.47
Sugar-sweetened beverages	8.19	0.46
Preserved and processed foods	7.34	0.22
Seafood	0.57	−0.31
Fruits	1.43	−0.30
Eggs	1.51	−0.30
Dark-colored vegetables	1.08	−0.24
Honey/jam	0.04	−0.19
Light-colored vegetables	0.47	−0.17
Sweet bread	7.76	−0.15
Whole grains	0.19	−0.12
Milk	5.59	−0.10
Fried rice/flour products	2.78	−0.09
Instant noodles	0.81	−0.07
Legumes/soy products	1.14	−0.07
Root crops	0.06	−0.04
Rice/flour products	0.02	−0.01
Dairy products	5.14	0.14
Organ meats	0.25	0.14
Meats	1.19	0.07
Vegetables with added oil/fats	<0.0001	0.07
Deep-fried foods	0.18	0.03

**Table 4:** Logistic regression models for the association of liver-associated dietary patterns and concordance lifestyle with abnormal liver function (*n* = 62,645).

Quartile	Dietary Pattern		Concordance Lifestyle ^a^	
	Model 1 ^b^	Model 2 ^c^	Model 1 ^b^	Model 2 ^c^
	OR (95% CI)	OR (95% CI)	OR (95% CI)	OR (95% CI)
1	1.00 (Ref)		1.00 (Ref)	
2	1.02 (0.99, 1.04)	1.03 (0.99, 1.07)	1.40 (1.30, 1.50)	1.23 (1.14, 1.32)
3	1.06 (1.03, 1.09)	1.07 (1.03, 1.11)	1.64 (1.53, 1.75)	1.29 (1.21, 1.39)
4	1.09 (1.06, 1.13)	1.08 (1.04, 1.12)	1.83 (1.70, 1.96)	1.42 (1.31, 1.53)
*p* for Trend	<0.001	0.001	<0.001	<0.001

^a^ Concordance lifestyle scores were calculated by computing all lifestyle variables and categorizing into quartiles. ^b^ Model 1 was adjusted for age and gender. ^c^ For dietary pattern, model 2 was adjusted for age, gender, marital status, education, family income, body mass index, body fat, systolic pressure, diastolic pressure, smoking status, drinking status, sleep duration, physical activity, and iron biomarkers. For concordance lifestyle, model 2 was adjusted for age, gender, marital status, education, family income, body mass index, body fat, systolic pressure, diastolic pressure, dietary categories, and iron biomarkers.

**Table 5:** Adjusted interaction models between dietary pattern and concordance lifestyle across the quartiles for the risk of abnormal liver function (*n* = 62,645) ^a^.

Quartiles of Dietary Pattern	Quartiles of Concordance Lifestyle			
	1	2	3	4
	OR (95% CI)	OR (95% CI)	OR (95% CI)	OR (95% CI)
1	1.00 (Ref)	1.25 (1.18, 1.32)	1.53 (1.46,1.61)	2.10 (1.99, 2.21)
2	1.08 (1.02, 1.14)	1.26 (1.19, 1.33)	1.56 (1.48, 1.65)	2.10 (1.99, 2.22)
3	1.09 (1.03, 1.15)	1.28 (1.21, 1.36)	1.61 (1.53, 1.70)	2.11 (1.98, 2.23)
4	1.21 (1.14, 1.28)	1.33 (1.26, 1.41)	1.63 (1.55, 1.72)	2.14 (2.02, 2.26)

^a^ Adjusted model for age, gender, marital status, education, family income, body mass index, body fat, systolic pressure, diastolic pressure, and iron biomarkers.

## Data Availability

Data were obtained from the Mei Jau Health Management Institution, but were restricted for research use only. The data are not publicly available.

## Supplementary Materials

The following are available online at https://www.mdpi.com/article/10.3390/nu13103591/s1, Table S1: Multivariate linear regression of liver function biomarkers across the quartiles of dietary pattern, Table S2: Logistic regression models for the association of lifestyle variables with abnormal liver function biomarkers (*n* = 62,645). Table S3: Multiple logistic regression for the association of dietary pattern or lifestyle variables with abnormal liver function biomarkers in both genders (*n* = 62,645).
