# Supplementary material for: Synergistic Interaction of Dietary Pattern and Concordance Lifestyle with Abnormal Liver Function among Young Adults in Taiwan: A Population-Based Longitudinal Study
Source: Nutrients. 2021 Oct 14;13(10):3591. doi: 10.3390/nu13103591 (PMC8539530; doi:10.3390/nu13103591)
Supplement: Supplementary file 1 [file nutrients-13-03591-s001.zip › nutrients-1380225-supplementary.pdf]

Table S1. Multivariate linear regression of liver function biomarkers across the quartiles of dietary pattern [a].

Liver Function Biomarkers	Quartiles of Dietary Pattern						
	1	2		3		4	
		β (95% CI)	*p*-Value	β (95% CI)	*p*-Value	β (95% CI)	*p*-Value
ALT (IU/L)	Ref	0.18 (0.06, 0.31)	0.000	0.31 (0.19, 0.44)	0.000	0.55 (0.42, 0.68)	0.000
AST (IU/L)		0.03 (-0.18, 0.24)	0.600	0.30 (0.09, 0.51)	0.000	0.47 (0.26, 0.68)	0.000
γ-GT (IU/L)		-0.03 (-0.18, 0.13)	0.671	0.12 (-0.03, 0.27)	0.363	-0.04 (-0.19, 0.11)	0.222
ALP (IU/L)		3.24 (2.38, 4.09)	0.000	4.91 (4.06, 5.76)	0.000	12.76 (11.90, 13.62)	0.000
LDH (IU/L)		2.81 (1.82, 3.80)	0.000	5.62 (4.63, 6.60)	0.000	13.59 (12.59, 14.58)	0.000
Albumin (g/dL)		-0.00 (-0.00, 0.01)	0.100	-0.01 (-0.01, -0.02)	0.001	-0.02 (-0.02, -0.01)	0.000
Total bilirubin (mg/dL)		0.01 (0.01, 0.02)	0.000	0.01 (0.01, 0.02)	0.000	0.01 (0.01, 0.02)	0.000

ALT: alanine transaminase, AST: aspartate transaminase, γ-GT gamma-glutamyltransferase, ALP: alkaline phosphatase, LDH: lactate dehydrogenase.

[a] Multivariate model was adjusted for age, gender, marital status, education, family income, body mass index, body fat, systolic pressure, diastolic pressure, smoking status, drinking status, sleep duration, and physical activity.

Table S2. Logistic regression models for the association of lifestyle variables with abnormal liver function biomarkers (n = 62,645).

Lifestyle Variables	Model 1 [a] OR (95% CI)	p-Value	Model 2 [b] OR (95% CI)	p-Value
Smoking status (Ref: no)				
Second-hand smoker	0.99 (0.94, 1.04)	0.599	0.97 (0.92, 1.02)	0.28
Past smoker	1.47 (1.38, 1.57)	<0.0001	1.43 (1.34, 1.52)	<0.0001
Smoke occasionally	1.28 (1.19, 1.37)	<0.0001	1.27 (1.18, 1.37)	<0.0001
Smoke daily	1.55 (1.49, 1.62)	<0.0001	1.54 (1.47, 1.61)	<0.0001
Drinking status (Ref: no)				
Past drinker	1.18 (1.05, 1.32)	0.005	1.03 (0.91, 1.17)	0.62
1-2 times/week	1.52 (1.46, 1.59)	<0.0001	1.39 (1.34, 1.46)	<0.0001
3-4 times/week	1.74 (1.62, 1.88)	<0.0001	1.57 (1.45, 1.70)	<0.0001
Drank daily	1.74 (1.54, 1.96)	<0.0001	1.57 (1.38, 1.78)	<0.0001
Sleep duration (Ref: normal)				
Short (<6 h)	1.12 (1.09, 1.15)	<0.0001	1.07 (1.04, 1.09)	<0.0001
Long (>8 h)	1.18 (1.09, 1.19)	<0.0001	1.11 (1.10, 1.12)	<0.0001
Physical activity (Ref: active)				
Less active (<2 h/week)	1.33 (1.26, 1.41)	<0.0001	1.25 (1.17, 1.33)	<0.0001
BMI (Ref: normal weight)				
Underweight	1.69 (1.62, 1.76)	<0.0001	1.71 (1.62, 1.80)	<0.0001
Overweight	3.30 (3.16, 3.45)	<0.0001	3.15 (2.96, 3.39)	<0.0001
Obese	4.75 (4.52, 4.99)	<0.0001	4.52 (4.23, 4.85)	<0.0001

BMI: body mass index.

[a] Model 1 was adjusted for age and gender.

[b] For smoking status, drinking status, sleep duration, and physical activity, model 2 was adjusted for age, gender, marital status, education, family income, body mass index, body fat, systolic pressure, and diastolic pressure. For BMI, model 2 was adjusted for age, gender, marital status, education, family income, body mass index, body fat, systolic pressure, diastolic pressure, smoking status, drinking status, sleep duration, and physical activity.

Table S3. Multiple logistic regression for the association of dietary pattern or lifestyle variables with abnormal liver function biomarkers in both genders (n = 62,645) [a].

Variables	OR (95% CI)			
	**Male (*n* = 13,189)**	***p*-Value**	**Female (*n* = 49,456)**	***p*-Value**
Liver associated dietary pattern (Ref: Q1)				
Q2	0.99 (0.97, 1.06)	0.087	1.09 (1.05, 1.12)	<0.0001
Q3	1.06 (0.92, 1.19)	0.210	1.14 (1.10, 1.18)	<0.0001
Q4	1.13 (1.08, 1.14)	0.014	1.18 (1.14, 1.19)	<0.0001
Smoking status (Ref: no)				
Second-hand smoker	1.13 (0.99, 1.26)	0.062	1.04 (0.89, 1.24)	0.610
Past smoker	1.19 (1.10, 1.28)	<0.0001	1.07 (1.06, 1.08)	0.002
Smoke occasionally	1.02 (0.92, 1.15)	0.639	1.08 (1.0.7, 1.09)	0.007
Smoke daily	0.97 (0.89, 1.06)	0.616	1.12 (1.09, 1.17)	<0.0001
Drinking status (Ref: no)				
Past drinker	1.07 (1.06, 1.09)	0.013	1.04 (0.89, 1.21)	0.584
1-2 times/week	1.08 (1.07, 1.08)	<0.0001	0.94 (0.88, 1.01)	0.095
3-4 times/week	1.09 (1.09, 1.11)	<0.0001	1.04 (1.01, 1.04)	<0.0001
Drank daily	1.11 (1.09, 1.16)	<0.0001	1.06 (1.05, 1.08)	<0.0001
Sleep duration (Ref: normal)				
Short (<6 h)	0.88 (0.84, 1.01)	0.083	1.01 (1.01, 1.02)	<0.0001
Long (>8 h)	1.11 (1.08, 1.29)	<0.0001	1.08 (1.02, 1.13)	0.002
Physical activity (Ref: active)				
Less active (<2 h/week)	1.31 (1.10, 1.55)	0.002	1.24 (1.03, 1.48)	0.020

[a] For smoking status, drinking status, sleep duration, and physical activity, age, marital status, education, family income, body mass index, body fat, systolic pressure, and diastolic pressure were adjusted.
